# Supplementary material for: End Sequence Analysis Toolkit (ESAT) expands the extractable information from single-cell RNA-seq data
Source: Genome Res. 2016 Oct;26(10):1397–410. doi: 10.1101/gr.207902.116 (PMC5052061; doi:10.1101/gr.207902.116)
Supplement: Supplemental Material [file supp_gr.207902.116_Supp_Materials.tar.gz › ESAT-esat_v0.1/doc/html/functions_s.html]

ESAT: Class Members


ESAT


- Main Page
- Related Pages
- Packages
- Classes
- Files

- Class List
- Class Index
- Class Hierarchy
- Class Members

- All
- Functions
- Variables
- Enumerator

- a
- b
- c
- d
- e
- f
- g
- h
- i
- j
- k
- l
- m
- n
- o
- p
- q
- r
- s
- t
- u
- v
- w
- z


All Classes Namespaces Files Functions Variables Enumerator Pages

Here is a list of all class members with links to the classes they belong to:

### - s -

- SameOrientationFilter()
  : umms.core.readFilters.SameOrientationFilter
- SAMFormatFullBED()
  : broad.pda.seq.alignment.AlignmentUtils
- SAMFragmentWriter()
  : broad.pda.samtools.SAMFragmentWriter
- SAMPairedEndFileReader()
  : broad.pda.samtools.SAMPairedEndFileReader
- sample()
  : broad.core.siphy.EvolutionaryModel
  , broad.pda.seq.protection.WindowScores
- sampleColumns()
  : broad.core.multiplealignment.MAFAlignment
  , broad.core.multiplealignment.MultipleAlignment
- SampleData()
  : broad.pda.seq.clip.SampleData
  , broad.pda.seq.protection.SampleData
- sampleModel
  : umms.core.model.score.MultiScore
- sampleName
  : broad.pda.seq.clip.SampleData
  , broad.pda.seq.protection.SampleData
- SAVED\_MODEL\_STATS\_EXT
  : broad.pda.seq.alignment.StoredAlignmentStats
- scaleNorm()
  : broad.core.datastructures.MatrixWithHeaders
- scaleRegion()
  : broad.core.siphy.TreeScaler
- scaleTree()
  : broad.core.siphy.TreeScaler
- scaleTreeWithSampling()
  : broad.core.siphy.TreeScaler
- scan()
  : broad.core.siphy.ConservedPWMScanner
  , broad.pda.seq.segmentation.ContinuousDataAlignmentModel
  , umms.core.annotation.AbstractAnnotationCollection< T extends Annotation >
  , umms.core.annotation.AnnotationCollection< T extends Annotation >
  , umms.core.model.AlignmentModel
  , umms.core.model.JCSAlignmentModel
- scanGenome()
  : broad.pda.seq.segmentation.ContinuousDataAlignmentModel
- scanPRate()
  : broad.pda.seq.segmentation.AlignmentDataModelStats
  , broad.pda.seq.segmentation.ContinuousDataAlignmentModel
- scanRegion()
  : broad.core.motif.PWMUtils
  , broad.pda.seq.segmentation.AlignmentDataModelStats
  , broad.pda.seq.segmentation.ContinuousDataAlignmentModel
- ScanStatisticScore()
  : umms.core.model.score.ScanStatisticScore
- score()
  : broad.core.siphy.PositionWeightMatrixModel
- SCORE
  : broad.pda.seq.rap.GenomeScoringProgram
  , broad.pda.seq.rap.rna.TranscriptomeScoringProgram
- scoreAnnotations()
  : broad.pda.seq.alignment.AlignmentUtils
- scoreGene()
  : broad.pda.seq.segmentation.ContinuousDataAlignmentModel
- scoreGenes()
  : broad.pda.seq.segmentation.ContinuousDataAlignmentModel
- scoreListByTStatistic()
  : broad.pda.geneexpression.ScoreDiffGenes
- ScoreMotifInstances()
  : broad.core.motif.misc.ScoreMotifInstances
- scoreMotifs()
  : broad.core.motif.misc.ComputeConservedMotifs
- scorePaths()
  : broad.pda.seq.segmentation.ContinuousDataAlignmentModel
- scorePathsForOnlyRPKM()
  : broad.pda.seq.segmentation.ContinuousDataAlignmentModel
- scoreSegment()
  : broad.pda.seq.segmentation.ContinuousDataAlignmentModel
- scoreSegments()
  : broad.pda.seq.segmentation.ContinuousDataAlignmentModel
- scoreWindow()
  : broad.pda.seq.protection.SampleData
- scoreWindowWithFragmentLengthFilter()
  : broad.pda.seq.protection.SampleData
- scramble()
  : broad.core.sequence.Sequence
- SearchException()
  : broad.core.motif.SearchException
- selectHighScoringIsoform()
  : broad.pda.gene.GeneTools
- SelectLongestIntronChainCandidate()
  : broad.pda.annotation.Locus
- selectRandIsoSubset()
  : broad.pda.annotation.Locus
  , broad.pda.gene.GeneWithIsoforms
- SELF
  : broad.pda.seq.graph.ChromosomeWithBubblesJGraphT.EdgeSourceType
- SEQ\_ID\_REC\_LENGTH
  : broad.core.sequence.FastaQualityBinaryIO
- Sequence()
  : broad.core.sequence.Sequence
  , jaligner.Sequence
- sequenceDirectory
  : broad.pda.feature.genome.DirectoryInstalledGenomeAssembly
- SequenceMotif()
  : broad.core.motif.SequenceMotif
- SequenceMotifCluster()
  : broad.core.motif.SequenceMotifCluster
- SequenceParserException()
  : jaligner.util.SequenceParserException
- SequenceRegion()
  : broad.core.sequence.SequenceRegion
- SequenceRegionWindow()
  : broad.core.sequence.SequenceRegionWindow
- set()
  : broad.core.datastructures.MatrixWithHeaders
  , jaligner.Cell
  , Jama.Matrix
- setA()
  : broad.core.annotation.BasicTwoSubjectAnnotation
- setAddToReadName()
  : broad.pda.seq.fastq.FastqSplitterByBarcode
- setAlignedSequences()
  : broad.core.multiplealignment.MAFAlignment
- setAlignment()
  : broad.core.siphy.TreeScaler
- setAlignmentChunkSize()
  : broad.core.siphy.ConservedPWMScanner
- setAlignmentDataModel()
  : broad.pda.seq.segmentation.AlignmentDataModelStats
- setAlignPos()
  : broad.core.motif.PositionWeightMatrix
- setAlpha()
  : broad.pda.seq.segmentation.ContinuousDataAlignmentModel
- setAlphabetSize()
  : broad.core.siphy.EvolutionaryModel
- setAnnotationList()
  : broad.core.annotation.BasicGenomicAnnotationReader
- setAsSafeToLoad()
  : broad.pda.seq.alignment.StoredAlignmentStats
- setAsUnsafeToLoad()
  : broad.pda.seq.alignment.StoredAlignmentStats
- setAtEnd()
  : broad.pda.seq.fastq.FastqSplitterByBarcode
- setAttribute()
  : umms.core.alignment.AbstractPairedEndAlignment
- setAttributes()
  : broad.core.annotation.GFF
- setAvergeNumberOfReads()
  : broad.pda.seq.segmentation.GeneScore
- setB()
  : broad.core.annotation.BasicTwoSubjectAnnotation
- setBackgroundModel()
  : broad.core.siphy.ConservedPWMScanner
- setBackgroundNucleotideFreqs()
  : broad.core.siphy.EvolutionaryModelParameters
- setBedScore()
  : broad.pda.gene.GeneWithIsoforms
  , umms.core.annotation.Gene
- setBinomialScore()
  : broad.pda.seq.protection.MultiSampleScanPeakCaller
- setBlastHit()
  : broad.core.primer3.HairpinKmer
- setBlocks()
  : broad.core.multiplealignment.MAFAlignment
- setBlockSizeStarts()
  : broad.core.annotation.BED
- setBoundariesFromAnnoations()
  : broad.core.annotation.BasicLightweightAnnotation
- setCapacity()
  : broad.core.sequence.Sequence
- setCDS()
  : umms.core.annotation.Gene
- setCDSEnd()
  : umms.core.annotation.Gene
- setCDSRegion()
  : umms.core.annotation.Gene
- setCDSStart()
  : umms.core.annotation.Gene
- setCharAt()
  : broad.core.sequence.Sequence
- setChromosome()
  : broad.core.annotation.BasicLightweightAnnotation
  , broad.core.annotation.LightweightGenomicAnnotation
  , broad.core.sequence.SequenceRegion
- setChromosomeLength()
  : broad.pda.seq.graph.ChromosomeWithBubblesJGraphT
- setChunkSize()
  : broad.pda.seq.segmentation.AlignmentDataModel
  , broad.pda.seq.segmentation.AlignmentDataModelStats
  , broad.pda.seq.segmentation.GenericAlignmentDataModel
- setClusterDistanceFunction()
  : broad.pda.geneexpression.clustering.HierarchicalClustering
- setCol()
  : jaligner.Cell
- setColumn()
  : broad.core.datastructures.MatrixWithHeaders
  , Jama.Matrix
- setColumns()
  : broad.pda.datastructures.LocationAwareMatrix
- setCoordinateSpace()
  : broad.pda.seq.protection.MultiSampleScanPeakCaller
- setCount()
  : umms.core.model.score.CountScore
  , umms.core.model.score.EndInWindowCountScore
  , umms.core.model.score.StartInWindowCountScore
- setCountNormalizer()
  : broad.pda.seq.segmentation.AlignmentDataModelStats
- setCountScore()
  : broad.pda.datastructures.Alignments
  , umms.core.annotation.Gene
- setCtrlCoordSpace()
  : umms.core.model.score.MultiScore
- setCtrlCount()
  : umms.core.model.score.BinomialEnrichmentScore
  , umms.core.model.score.MultiScore
  , umms.core.model.score.PoissonEnrichmentScore
- setCtrlRegionCount()
  : umms.core.model.score.BinomialEnrichmentScore
  , umms.core.model.score.MultiScore
- setCtrlRegionTotal()
  : umms.core.model.score.PoissonEnrichmentScore
- setCtrlTotal()
  : umms.core.model.score.PoissonEnrichmentScore
- setCurrentSequenceId()
  : broad.core.sequence.AbstractFastaParser
- setData()
  : broad.core.datastructures.MatrixWithHeaders
- setDataDimensions()
  : broad.pda.datastructures.LocationAwareMatrix
- setDenominatorCount()
  : umms.core.model.score.NewRatioScore
  , umms.core.model.score.RatioScore
  , umms.core.model.score.WindowAllScore
- setDenominatorRegionTotal()
  : umms.core.model.score.NewRatioScore
  , umms.core.model.score.RatioScore
  , umms.core.model.score.WindowAllScore
- setDenominatorScanPVal()
  : umms.core.model.score.WindowAllScore
- setDenominatorTotal()
  : umms.core.model.score.NewRatioScore
  , umms.core.model.score.RatioScore
  , umms.core.model.score.WindowAllScore
- setDescription()
  : broad.pda.seq.fastq.FastqSequence
  , jaligner.Sequence
- setDiagonal()
  : jaligner.TracebackNode
- setDuplicateFlag()
  : umms.core.alignment.AbstractPairedEndAlignment
  , umms.core.alignment.Alignment
  , umms.core.alignment.SingleEndAlignment
- setDuplicateReadFlag()
  : umms.core.alignment.SingleEndAlignment.WrapSamRecord
- setEnd()
  : broad.core.annotation.LightweightGenomicAnnotation
  , broad.core.sequence.SequenceRegion
  , broad.pda.seq.graph.ChromosomeWithBubblesJGraphT
  , umms.core.annotation.Annotation
  , umms.core.annotation.BasicAnnotation
  , umms.core.annotation.CompoundInterval
- setEndStateTransitionProbability()
  : broad.core.hmm.MarkovModel< T >
- setEnrichment()
  : broad.pda.seq.segmentation.GeneScore
- setEstimationWindow()
  : broad.core.siphy.TreeScalerIO
- setExonScores()
  : broad.pda.seq.graph.ChromosomeWithBubblesJGraphT
- setExpressionScanPvalueCutoff()
  : broad.pda.seq.clip.SampleData
  , broad.pda.seq.protection.MultiSampleScanPeakCaller
  , broad.pda.seq.protection.SampleData
- setExtend()
  : jaligner.Alignment
- setExtensionFactor()
  : broad.pda.seq.segmentation.AlignmentDataModel
  , broad.pda.seq.segmentation.AlignmentDataModelStats
  , broad.pda.seq.segmentation.ContinuousDataAlignmentModel
  , broad.pda.seq.segmentation.GenericAlignmentDataModel
- setExtraFields()
  : broad.pda.gene.GeneWithIsoforms
  , broad.pda.seq.protection.MultiSampleScanPeakCaller
  , umms.core.annotation.Gene
- setFeature()
  : broad.core.annotation.GFF
- setFilterByStrand()
  : broad.pda.seq.protection.MultiSampleScanPeakCaller
- setFindMaxContiguous()
  : broad.pda.seq.segmentation.ContinuousDataAlignmentModel
- setFirstRead()
  : broad.pda.seq.segmentation.AlignmentDataModel
  , broad.pda.seq.segmentation.GenericAlignmentDataModel
- setFirstReadTranscriptionStrand()
  : broad.pda.seq.protection.MultiSampleScanPeakCaller
- setFivePrimeBuffer()
  : broad.core.annotation.BasicGenomicAnnotation
- setForwardStrand()
  : broad.core.sequence.Sequence
- setFragmentFlag()
  : umms.core.readers.PairedEndReader
- setFragmentStrand()
  : umms.core.alignment.AbstractPairedEndAlignment
  , umms.core.alignment.Alignment
  , umms.core.alignment.SingleEndAlignment
- setFrame()
  : broad.core.annotation.GFF
- setFromCounts()
  : broad.core.motif.PositionWeightColumn
- setFullyContainedNumberOfReads()
  : broad.pda.seq.segmentation.GeneScore
  , umms.core.model.score.BasicScanStatisticScore
  , umms.core.model.score.ScanStatisticScore
- setGaps()
  : jaligner.Alignment
- setGene()
  : umms.core.model.score.MultiScore
- setGeneLength()
  : broad.pda.seq.segmentation.GeneScore
- setGlobalLength()
  : umms.core.model.score.BasicScanStatisticScore
  , umms.core.model.score.PoissonEnrichmentScore
  , umms.core.model.score.ScanStatisticScore
- setGroup()
  : broad.core.annotation.GFF
- setHasIndel()
  : umms.core.alignment.SingleEndAlignment
- setHeader()
  : umms.core.alignment.AbstractPairedEndAlignment
  , umms.core.alignment.Alignment
  , umms.core.alignment.SingleEndAlignment
- setId()
  : broad.core.annotation.BasicGenomicAnnotation
  , broad.core.annotation.GenomicAnnotation
  , broad.core.sequence.Sequence
  , broad.core.sequence.SequenceRegion
  , jaligner.formats.Format
  , jaligner.Sequence
- setIdentity()
  : jaligner.Alignment
- setIgnoreList()
  : broad.core.siphy.ConservedPWMScanner
- setInitialStateTransitionProbability()
  : broad.core.hmm.MarkovModel< T >
- setIOHelper()
  : broad.core.multiplealignment.MultipleAlignment
- setIsFirstMate()
  : umms.core.alignment.SingleEndAlignment
- setIsToComplex()
  : broad.pda.seq.graph.Path
- setJnlp()
  : jaligner.util.Commons
- setLambda()
  : broad.pda.seq.segmentation.AlignmentDataModelStats
- setLeft()
  : jaligner.TracebackNode
- setLine()
  : broad.pda.datastructures.Alignments
- setLinkage()
  : broad.pda.geneexpression.clustering.HierarchicalClustering
- setLocalLambda()
  : broad.pda.seq.graph.Path
  , broad.pda.seq.segmentation.GeneScore
- setLocalRate()
  : broad.pda.seq.graph.ChromosomeWithBubblesJGraphT
- setLoggerLevel()
  : broad.pda.seq.protection.MultiSampleScanPeakCaller
- setMarkupLine()
  : jaligner.Alignment
- setMaskFileData()
  : broad.pda.seq.segmentation.ContinuousDataAlignmentModel
- setMatch()
  : umms.core.annotation.PSLRecord
- setMatrix()
  : broad.core.motif.PositionWeightMatrix
  , jaligner.Alignment
  , Jama.Matrix
- setMatrixFromRawData()
  : broad.core.motif.PositionWeightMatrix
- setMaxAllowableInsert()
  : umms.core.writers.PairedEndWriter
- setMinContguousSegmentSize()
  : broad.pda.seq.segmentation.ContinuousDataAlignmentModel
- setMinimumMappingQuality()
  : broad.pda.seq.segmentation.AlignmentDataModel
  , broad.pda.seq.segmentation.AlignmentDataModelStats
  , broad.pda.seq.segmentation.ContinuousDataAlignmentModel
  , broad.pda.seq.segmentation.GenericAlignmentDataModel
- setMinimumTreeLength()
  : broad.core.siphy.TreeScaler
- setMinNumberOfSplices()
  : broad.pda.seq.segmentation.ContinuousDataAlignmentModel
- setMirScore()
  : broad.core.primer3.HairpinKmer
- setMismatch()
  : umms.core.annotation.PSLRecord
- setModel()
  : broad.core.siphy.ConservedPWMScanner
  , broad.core.siphy.TreeScaler
- setModelParameters()
  : broad.core.siphy.EvolutionaryModel
- setMotif()
  : broad.core.motif.SequenceMotif
- setName()
  : broad.core.annotation.LightweightGenomicAnnotation
  , broad.core.motif.PositionWeightMatrix
  , broad.core.sequence.SequenceRegion
  , broad.pda.seq.fastq.FastqSequence
  , umms.core.annotation.Annotation
  , umms.core.annotation.BasicAnnotation
- setName1()
  : jaligner.Alignment
- setName2()
  : jaligner.Alignment
- setNcount()
  : umms.core.annotation.PSLRecord
- setNegativeStranded()
  : broad.pda.seq.segmentation.AlignmentDataModel
  , broad.pda.seq.segmentation.GenericAlignmentDataModel
- setNeutralModel()
  : broad.core.siphy.TreeScaler
- setNeutralOmegaDistribution()
  : broad.core.siphy.EvolutionaryModel
- setNominalPValue()
  : broad.pda.seq.segmentation.GeneScore
- setNormalizationStrategy()
  : broad.pda.seq.segmentation.AlignmentDataModel
  , broad.pda.seq.segmentation.GenericAlignmentDataModel
- setNum()
  : broad.core.motif.PositionWeightColumn
- setNumberFormatter()
  : broad.core.siphy.TreeScalerIO
- setNumberOfMarkers()
  : broad.pda.seq.segmentation.AlignmentDataModelStats
- setNumberOfReads()
  : broad.pda.seq.segmentation.AlignmentDataModelStats
  , broad.pda.seq.segmentation.GeneScore
- setNumeratorCount()
  : umms.core.model.score.NewRatioScore
  , umms.core.model.score.RatioScore
  , umms.core.model.score.WindowAllScore
- setNumeratorRegionTotal()
  : umms.core.model.score.NewRatioScore
  , umms.core.model.score.RatioScore
  , umms.core.model.score.WindowAllScore
- setNumeratorScanPVal()
  : umms.core.model.score.WindowAllScore
- setNumeratorTotal()
  : umms.core.model.score.NewRatioScore
  , umms.core.model.score.RatioScore
  , umms.core.model.score.WindowAllScore
- setOmega()
  : broad.core.siphy.EvolutionaryModel
- setOmegaDistByTreeLength()
  : broad.core.siphy.EvolutionaryModel
- setOpen()
  : jaligner.Alignment
- setOrientation()
  : broad.core.annotation.BasicGenomicAnnotation
  , broad.core.annotation.BasicLightweightAnnotation
  , broad.core.annotation.GenomicAnnotation
  , broad.core.annotation.LightweightGenomicAnnotation
  , broad.core.sequence.SequenceRegion
  , umms.core.annotation.AbstractAnnotation
  , umms.core.annotation.Annotation
  , umms.core.annotation.BasicAnnotation
  , umms.core.annotation.Gene
- setOrientedEnd()
  : broad.core.sequence.SequenceRegion
  , umms.core.annotation.Annotation
  , umms.core.annotation.BasicAnnotation
- setOrientedStart()
  : broad.core.sequence.SequenceRegion
  , umms.core.annotation.Annotation
  , umms.core.annotation.BasicAnnotation
- setOriginalScore()
  : broad.core.primer3.HairpinKmer
- setOriginalSequence1()
  : jaligner.Alignment
- setOriginalSequence2()
  : jaligner.Alignment
- setPair1Parser()
  : broad.pda.seq.fastq.FastqSplitterByBarcode
- setPair2Parser()
  : broad.pda.seq.fastq.FastqSplitterByBarcode
- setPaired()
  : broad.pda.seq.fastq.FastqSplitterByBarcode
- setParameters()
  : broad.core.siphy.EvolutionaryModel
- setPeakCutoffMostCommonReplicate()
  : broad.pda.seq.protection.MultiSampleScanPeakCaller
- setPeakTrimQuantile()
  : broad.pda.seq.protection.MultiSampleScanPeakCaller
- setPeakWindowCountCutoff()
  : broad.pda.seq.protection.MultiSampleScanPeakCaller
- setPeakWindowScanPvalCutoff()
  : broad.pda.seq.protection.MultiSampleScanPeakCaller
- setPercentMaskedAllowed()
  : umms.core.coordinatesystem.GenomicSpace
  , umms.core.coordinatesystem.TranscriptInGenomicSpace
- setPercentVariantsCovered()
  : broad.core.primer3.HairpinKmer
- setPi()
  : broad.core.siphy.EvolutionaryModel
- setPIDToName()
  : broad.core.datastructures.MatrixWithHeaders
- setPositiveStranded()
  : broad.pda.seq.segmentation.AlignmentDataModel
  , broad.pda.seq.segmentation.GenericAlignmentDataModel
- setProgramDescription()
  : broad.core.parser.CommandLineParser
- setProperPairFlag()
  : umms.core.alignment.AbstractPairedEndAlignment
  , umms.core.alignment.Alignment
  , umms.core.alignment.SingleEndAlignment
- setPvalue()
  : umms.core.model.score.BasicScanStatisticScore
  , umms.core.model.score.BinomialEnrichmentScore
  , umms.core.model.score.PoissonEnrichmentScore
  , umms.core.model.score.ScanStatisticScore
- setQueryEnd()
  : umms.core.annotation.PSLRecord
- setQueryGap()
  : umms.core.annotation.PSLRecord
- setQueryGapBases()
  : umms.core.annotation.PSLRecord
- setQuerySize()
  : umms.core.annotation.PSLRecord
- setQueryStart()
  : umms.core.annotation.PSLRecord
- setRateMatrix()
  : broad.core.siphy.EvolutionaryModelParameters
- setRead1Trim3p()
  : broad.pda.seq.fastq.FastqSplitterByBarcode
- setRead1Trim5p()
  : broad.pda.seq.fastq.FastqSplitterByBarcode
- setRead2Trim3p()
  : broad.pda.seq.fastq.FastqSplitterByBarcode
- setRead2Trim5p()
  : broad.pda.seq.fastq.FastqSplitterByBarcode
- setReasonForUnsafe()
  : broad.pda.seq.alignment.StoredAlignmentStats
- setReferenceId()
  : broad.core.multiplealignment.MultipleAlignment
- setReferenceName()
  : broad.core.sequence.SequenceRegion
  , umms.core.annotation.Annotation
  , umms.core.annotation.BasicAnnotation
- setRefGapped()
  : broad.core.multiplealignment.MultipleAlignment
- setRegionEnd()
  : broad.core.sequence.SequenceRegion
- setRegionLength()
  : umms.core.model.score.BasicScanStatisticScore
  , umms.core.model.score.BinomialEnrichmentScore
  , umms.core.model.score.MultiScore
  , umms.core.model.score.PoissonEnrichmentScore
  , umms.core.model.score.ScanStatisticScore
- setRegionStart()
  : broad.core.sequence.SequenceRegion
- setRegionTotal()
  : umms.core.model.score.CountScore
- setRemoveDuplicatesFlag()
  : broad.pda.seq.segmentation.GenericAlignmentDataModel
- setRepMatch()
  : umms.core.annotation.PSLRecord
- setReversedOrientation()
  : broad.core.annotation.BasicLightweightAnnotation
  , broad.core.annotation.GenomicAnnotation
  , broad.core.sequence.SequenceRegion
- setRgb()
  : broad.core.annotation.BED
- setRow()
  : broad.core.datastructures.MatrixWithHeaders
  , jaligner.Cell
  , Jama.Matrix
- setRowDescription()
  : broad.core.datastructures.MatrixWithHeaders
- setRPKM()
  : broad.pda.seq.segmentation.GeneScore
- setRPKMConstant()
  : broad.pda.seq.graph.ChromosomeWithBubblesJGraphT
- setRS8Score()
  : broad.core.primer3.HairpinKmer
- setSampleCoordSpace()
  : umms.core.model.score.MultiScore
- setSampleCount()
  : umms.core.model.score.BinomialEnrichmentScore
  , umms.core.model.score.MultiScore
  , umms.core.model.score.PoissonEnrichmentScore
- setSampleName()
  : broad.pda.seq.clip.SampleData
  , broad.pda.seq.protection.SampleData
- setSampleRegionCount()
  : umms.core.model.score.BinomialEnrichmentScore
  , umms.core.model.score.MultiScore
- setSampleRegionTotal()
  : umms.core.model.score.PoissonEnrichmentScore
- setSampleTotal()
  : umms.core.model.score.PoissonEnrichmentScore
- setSAMString()
  : umms.core.annotation.Gene
- setScanPvalue()
  : broad.pda.seq.segmentation.GeneScore
  , umms.core.model.score.BasicScanStatisticScore
  , umms.core.model.score.ScanStatisticScore
- setScore()
  : broad.core.annotation.BasicLightweightAnnotation
  , broad.core.annotation.LightweightGenomicAnnotation
  , broad.core.multiplealignment.MultipleAlignment
  , broad.core.sequence.SequenceRegion
  , broad.core.sequence.SequenceRegionWindow
  , broad.pda.geneexpression.clustering.Cluster
  , jaligner.Alignment
  , jaligner.Cell
  , umms.core.annotation.Annotation
  , umms.core.annotation.BasicAnnotation
- setScores()
  : broad.pda.datastructures.Alignments
  , umms.core.model.score.AbstractListScore
- setSecondRead()
  : broad.pda.seq.segmentation.AlignmentDataModel
  , broad.pda.seq.segmentation.GenericAlignmentDataModel
- setSeed()
  : umms.core.coordinatesystem.GenomicSpace
  , umms.core.coordinatesystem.TranscriptInGenomicSpace
- setSentinel()
  : broad.core.datastructures.IntervalTree< V >
- setSequence()
  : broad.core.annotation.BasicGenomicAnnotation
  , broad.core.annotation.GenomicAnnotation
  , broad.core.sequence.SequenceRegion
  , jaligner.Sequence
  , umms.core.annotation.Gene
- setSequence1()
  : jaligner.Alignment
- setSequence2()
  : jaligner.Alignment
- setSequenceBases()
  : broad.core.sequence.Sequence
- setSequenceFile()
  : broad.pda.feature.genome.Chromosome
- setSequenceFromChromosome()
  : umms.core.annotation.Gene
- setSimilarity()
  : jaligner.Alignment
- setSource()
  : broad.core.annotation.GFF
  , broad.core.sequence.FastaSequenceIO
- setSpacing()
  : broad.core.multiplealignment.PhylipInterleavedMultipleAlignmentIO
- setSplicedEdges()
  : umms.core.alignment.SingleEndAlignment
- setSpliceWeight()
  : broad.pda.seq.graph.ChromosomeWithBubblesJGraphT
  , broad.pda.seq.graph.Path
- setStart()
  : broad.core.annotation.BasicLightweightAnnotation
  , broad.core.annotation.LightweightGenomicAnnotation
  , broad.core.sequence.SequenceRegion
  , broad.pda.seq.graph.ChromosomeWithBubblesJGraphT
  , umms.core.annotation.Annotation
  , umms.core.annotation.BasicAnnotation
  , umms.core.annotation.CompoundInterval
- setStart1()
  : jaligner.Alignment
- setStart2()
  : jaligner.Alignment
- setStateTransitionProbability()
  : broad.core.hmm.MarkovModel< T >
- setStop()
  : jaligner.TracebackNode
- setStoredStats()
  : broad.pda.seq.segmentation.AlignmentDataModelStats
- setStrand()
  : broad.core.annotation.GFF
- setSubcluster()
  : broad.pda.geneexpression.clustering.Cluster
- setTargetGap()
  : umms.core.annotation.PSLRecord
- setTargetGapBases()
  : umms.core.annotation.PSLRecord
- setTargetSize()
  : umms.core.annotation.PSLRecord
- setThickEnd()
  : broad.core.annotation.BED
- setThickStart()
  : broad.core.annotation.BED
- setThreePrimeBuffer()
  : broad.core.annotation.BasicGenomicAnnotation
- setTotal()
  : umms.core.model.score.CountScore
- setTree()
  : broad.core.siphy.EvolutionaryModelParameters
- setTrimEnds()
  : broad.pda.seq.segmentation.ContinuousDataAlignmentModel
- setTrimQuantile()
  : broad.pda.seq.segmentation.ContinuousDataAlignmentModel
- setType()
  : jaligner.Sequence
- setUninformativeNodes()
  : broad.core.siphy.ConservationUtils
- setUnparsedRow()
  : broad.core.motif.PositionWeightMatrix
- setup()
  : broad.pda.seq.rap.rna.CollectRnaSeqMetrics
- setUp()
  : jaligner.TracebackNode
- setUpAlignment()
  : broad.core.siphy.ConservationUtils
- setUpMAF()
  : broad.core.siphy.ConservationUtils
- setUpWeightSplices()
  : broad.pda.seq.segmentation.ContinuousDataAlignmentModel
- setValue1()
  : broad.core.datastructures.Pair< T1 >
- setValue2()
  : broad.core.datastructures.Pair< T1 >
- setWeighReadCountsFlag()
  : broad.pda.seq.segmentation.GenericAlignmentDataModel
- setWindowLength()
  : umms.core.model.score.BasicScanStatisticScore
- shift()
  : broad.core.annotation.AnnotationReader< T extends GenomicAnnotation >
  , broad.core.sequence.SequenceRegion
  , broad.core.siphy.StationaryDistributionIO
  , umms.core.alignment.AbstractPairedEndAlignment
  , umms.core.annotation.Annotation
  , umms.core.annotation.BasicAnnotation
  , umms.core.annotation.CompoundInterval
- shiftPosition()
  : broad.core.sequence.TranscribedRegions
- SHORT\_ENCODED\_a
  : broad.core.sequence.Sequence
- SHORT\_ENCODED\_A
  : broad.core.sequence.Sequence
- SHORT\_ENCODED\_c
  : broad.core.sequence.Sequence
- SHORT\_ENCODED\_C
  : broad.core.sequence.Sequence
- SHORT\_ENCODED\_g
  : broad.core.sequence.Sequence
- SHORT\_ENCODED\_G
  : broad.core.sequence.Sequence
- SHORT\_ENCODED\_GAP
  : broad.core.sequence.Sequence
- SHORT\_ENCODED\_N
  : broad.core.sequence.Sequence
- SHORT\_ENCODED\_T
  : broad.core.sequence.Sequence
- SHORT\_ENCODED\_t
  : broad.core.sequence.Sequence
- SHORT\_READ\_FLOW
  : broad.core.sequence.Sequence
- ShortBED()
  : broad.core.annotation.ShortBED
- shortBEDFactory
  : broad.core.annotation.AnnotationFactoryFactory
- ShortBEDReader()
  : broad.core.annotation.ShortBEDReader
- shuffle()
  : broad.core.math.MathUtil
  , broad.core.sequence.Sequence
  , broad.pda.feature.genome.Chromosome
- shuffleAndWriteAnnotations()
  : broad.pda.seq.rap.ShuffleBED
- ShuffledIterator()
  : umms.core.coordinatesystem.ShuffledIterator< T extends Annotation >
- shufflePeaks()
  : broad.pda.seq.rap.CollectAnnotationEnrichments
- shuffleScores()
  : broad.core.annotation.AnnotationReader< T extends GenomicAnnotation >
- shuffleWithinValid()
  : broad.pda.feature.genome.Chromosome
- signalData
  : broad.pda.seq.protection.TwoSampleScanSkellamPeakCaller
- signalName
  : broad.pda.seq.protection.TwoSampleScanSkellamPeakCaller
- signalProcessor
  : broad.pda.seq.protection.TwoSampleScanSkellamPeakCaller
- signalSamples
  : broad.pda.seq.protection.MultiSampleScanPeakCaller
- SIMILARITY
  : jaligner.Markups
- SIMPLE\_ARRAY\_LEFT
  : broad.pda.seq.rap.CountProbes
- SIMPLE\_ARRAY\_RIGHT
  : broad.pda.seq.rap.CountProbes
- SingleEndAlignment()
  : umms.core.alignment.SingleEndAlignment
- SingleInterval()
  : umms.core.annotation.SingleInterval
- SingularValueDecomposition()
  : Jama.SingularValueDecomposition
- size()
  : broad.core.annotation.AnnotationReader< T extends GenomicAnnotation >
  , broad.core.datastructures.IntervalTree< V >
  , broad.core.sequence.SequenceRegion
- SIZE
  : jaligner.matrix.Matrix
- size()
  : umms.core.annotation.AbstractAnnotation
  , umms.core.annotation.Annotation
  , umms.core.annotation.AnnotationCollection< T extends Annotation >
  , umms.core.annotation.AnnotationList< T extends Annotation >
  , umms.core.model.AlignmentModel
  , umms.core.model.JCSAlignmentModel
- sizeFile
  : broad.pda.seq.protection.MultiSampleScanPeakCaller
- SIZES
  : broad.pda.seq.rap.GenomeCommandLineProgram
- skellamCdf()
  : broad.core.math.Distribution
- skellamDensity()
  : broad.core.math.Distribution
- skellamLeftTail()
  : broad.core.math.Distribution
- skellamPvalue()
  : broad.core.math.Distribution
- skellamRightTail()
  : broad.core.math.Distribution
- SkellamScore()
  : broad.pda.seq.rap.SkellamScore
- slidePWM()
  : broad.core.siphy.ConservedPWMScanner
  , broad.core.siphy.TreeScaler
- slideWindowComputeOmega()
  : broad.core.siphy.tools.conservation.EstimateOmegaPerExon
- SmatchLike()
  : umms.core.alignment.SmatchLike
- SmithWatermanAlignment()
  : umms.core.alignment.SmithWatermanAlignment
- solve()
  : Jama.CholeskyDecomposition
  , Jama.LUDecomposition
  , Jama.Matrix
  , Jama.QRDecomposition
- solveTranspose()
  : Jama.Matrix
- SORT\_ORDER
  : broad.pda.samtools.FilterPairedReadsByMAQ
- sortColumns()
  : Jama.Matrix
- sortColumnsKeepingIndex()
  : Jama.Matrix
- sortList()
  : broad.core.datastructures.MatrixWithHeaders
- source
  : broad.core.annotation.BasicGenomicAnnotationReader
- spearmanCorrelation()
  : broad.core.math.Statistics
- SPLICED
  : broad.pda.seq.graph.ChromosomeWithBubblesJGraphT.EdgeSourceType
- SplitAndTrimIndex()
  : broad.pda.seq.fastq.SplitAndTrimIndex
- splitByChr()
  : broad.pda.rnai.ExtractSequence
- SplitByName()
  : broad.pda.differentialExpression.SplitByName
- SplitFastqByIndex()
  : broad.pda.seq.fastq.SplitFastqByIndex
- SplitFile()
  : broad.core.siphy.tools.conservation.SplitFile
- standardizeOreintation()
  : broad.pda.gene.GeneWithIsoforms
- start()
  : broad.pda.seq.fastq.FastqParser
  , broad.pda.seq.fastq.FastqSplitterByBarcode
- START\_CODON\_PATRN
  : umms.core.annotation.Gene
- StartInWindowCountScore()
  : umms.core.model.score.StartInWindowCountScore
- startTrack()
  : broad.core.annotation.AnnotationReader< T extends GenomicAnnotation >
- StationaryDistributionIO()
  : broad.core.siphy.StationaryDistributionIO
- statistic
  : broad.pda.enrichment.EnrichmentMetric
- statisticName
  : broad.pda.enrichment.EnrichmentMetric
- STATS
  : broad.pda.seq.rap.CountProbes
- stdev()
  : broad.core.math.Statistics
- stepSize
  : broad.pda.seq.protection.MultiSampleScanPeakCaller
  , broad.pda.seq.protection.SampleData
  , broad.pda.seq.protection.TwoSampleScanSkellamPeakCaller
  , broad.pda.seq.protection.WindowScores
- stitch()
  : broad.core.siphy.TreeScalerIO
- stitchList()
  : broad.core.annotation.BasicLightweightAnnotation
- stitchTo()
  : broad.core.annotation.BasicLightweightAnnotation
  , broad.core.annotation.LightweightGenomicAnnotation
  , broad.core.sequence.SequenceRegion
  , umms.core.annotation.Annotation
  , umms.core.annotation.BasicAnnotation
- stop()
  : broad.pda.seq.fastq.FastqSplitterByBarcode
- STOP
  : jaligner.Directions
- STOP\_CODONS
  : umms.core.annotation.Gene
- store()
  : broad.pda.seq.alignment.StoredAlignmentStats
- StoredAlignmentStats()
  : broad.pda.seq.alignment.StoredAlignmentStats
- StringParser()
  : broad.core.parser.StringParser
- submatrixByColumnIndex()
  : broad.core.datastructures.MatrixWithHeaders
- submatrixByColumnNames()
  : broad.core.datastructures.MatrixWithHeaders
- submatrixByColumns()
  : broad.pda.datastructures.LocationAwareMatrix
- submatrixByRowDescriptions()
  : broad.core.datastructures.MatrixWithHeaders
- submatrixByRowNames()
  : broad.core.datastructures.MatrixWithHeaders
- submitJobs()
  : broad.pda.seq.rap.CollectAnnotationEnrichments
- subsequence()
  : jaligner.Sequence
- sum()
  : broad.core.math.Statistics
- SumScore()
  : umms.core.model.score.SumScore
- svd()
  : Jama.Matrix
- SYMMETRIC
  : broad.pda.seq.rap.PlotAggregateRegions


---

Generated on Fri Aug 15 2014 15:11:16 for ESAT by  

 1.8.7
